# Supplementary material for: MetaRibo-Seq measures translation in microbiomes
Source: Nat Commun. 2020 Jun 29;11:3268. doi: 10.1038/s41467-020-17081-z (PMC7324362; doi:10.1038/s41467-020-17081-z)
Supplement: Supplementary file 10 — Supplementary Data 7 [file 41467_2020_17081_MOESM10_ESM.zip › File2/Confidence_High_Taxonomy/110419_out.krona.html]

Javascript must be enabled to view this page.

members
magnitude
magnitudeUnassigned
count
unassigned
taxon
rank

110419\_out

4

2
4
superkingdom

1239
4
phylum

class
4
186801

order
4
186802

4
family
990719


SRS142599\_contig\_number\_18697
1
species
1930012

1930013
species
3

SRS014235\_contig\_number\_contig-100\_1653.245242SRS024132\_contig\_number\_31522SRS104311\_contig\_number\_6674
